# Ibrutinib impairs IGF-1-dependent activation of intracellular Ca handling in isolated mouse ventricular myocytes

**DOI:** 10.3389/fcvm.2023.1190099

**Published:** 2023-08-15

**Authors:** Daniel Tarnowski, Anna-Lena Feder, Maximilian Trum, Klaus-Georg Kreitmeier, Laura Stengel, Lars S. Maier, Can Martin Sag

**Affiliations:** ^1^ Department of Internal Medicine II/Cardiology, University Medical Center Regensburg, Regensburg, Germany; ^2^ Department of Internal Medicine III/Oncology, University Medical Center Regensburg, Regensburg, Germany

**Keywords:** Ibrutinib, IGF-1, EC-coupling, heart failure, SR Ca handling

## Abstract

**Background:**

The Bruton tyrosine kinase (BTK) inhibitor Ibrutinib is associated with a higher incidence of cardiotoxic side effects including heart failure (HF).

**Objectives:**

Ibrutinib is capable of inhibiting PI3K/Akt signaling in neonatal rat ventricular cardiomyocytes when stimulated with insulin-like growth factor 1 (IGF-1). We therefore hypothesized that Ibrutinib might disrupt IGF-1-mediated activation of intracellular Ca handling in adult mouse cardiomyocytes by inhibiting PI3K/Akt signaling.

**Methods:**

Isolated ventricular myocytes (C57BL6/J) were exposed to IGF-1 at 10 nmol/L in the presence or absence of Ibrutinib (1 µmol/L) or Acalabrutinib (10 µmol/L; cell culture for 24 ± 2 h). Intracellular Ca handling was measured by epifluorescence (Fura-2 AM) and confocal microscopy (Fluo-4 AM). Ruptured-patch whole-cell voltage-clamp was used to measure *I*_Ca_. Levels of key cardiac Ca handling proteins were investigated by immunoblots.

**Results:**

IGF-1 significantly increased Ca transient amplitudes by ∼83% as compared to vehicle treated control cells. This was associated with unaffected diastolic Ca, enhanced SR Ca loading and increased *I*_Ca_. Co-treatment with Ibrutinib attenuated both the IGF-1-mediated increase in SR Ca content and in *I*_Ca_. IGF-1 treated cardiomyocytes had significantly increased levels of pS473Akt/Akt and SERCA2a expression as compared to cells concomitantly treated with IGF-1 and Ibrutinib. SR Ca release (as assessed by Ca spark frequency) was unaffected by either treatment. In order to test for potential off-target effects, second generation BTK inhibitor Acalabrutinib with greater BTK selectivity and lower cardiovascular toxicity was tested for IGF1-mediated activation of intracellular Ca handling. Acalabrutinib induced similar effects on Ca handling in IGF-1 treated cultured myocytes as Ibrutinib in regard to decreased Ca transient amplitude and slowed Ca transient decay, hence implying a functional class effect of BTK inhibitors in cardiac myocytes.

**Conclusions:**

Inhibition of BTK by Ibrutinib impairs IGF-1-dependent activation of intracellular Ca handling in adult ventricular mouse myocytes in the face of disrupted Akt signaling and absent SERCA2a upregulation.

## Introduction

The Bruton Tyrosin Kinase (BTK)-Inhibitor, Ibrutinib, is an established therapeutic agent for patients with B-cell malignancies such as relapsed or refractory chronic lymphocytic leukemia (CLL) (1, 2), mantle cell lymphoma (MCL) (3), and Waldenström macroglobulinemia (4). However, even though Ibrutinib is generally well tolerated, adverse events including cardiotoxicity can have substantial impact on the patients' health and can lead to therapy discontinuation eventually. For example, a 10-fold increase in new-onset of atrial fibrillation (AF) was observed in patients receiving Ibrutinib compared to Ofatumumab as the randomized RESONATE study outlined (1). More recently, Salem et al. reported a so far underappreciated yet higher incidence of heart failure (HF) in individuals treated with Ibrutinib (5). However, the underlying molecular mechanisms by which Ibrutinib might affect cardiac contractility remain largely unclear.

BTK plays a key role in promoting different intracellular activation pathways, such as Akt/PKB (Proteinkinase B) which is a downstream effector of phosphoinositide 3-kinase (PI3K) (6), and acts as a regulator for CLL-cell survival and proliferation (7). Only recent studies suggest that PI3K/Akt-signaling seems to play a pivotal role in the homeostatic regulation of calcium (Ca) in the cardiovascular system (6). Mouse experiments show that this pathway is a crucial mediator of cardiac protection under stress conditions (8). Moreover, increased activity of PI3K-Akt-signaling rescues cardiac dysfunction in murine models of heart failure (9). Class IA PI3K(p110α), the cardiac-protective isoform of PI3K, acts as a master effector of insulin-like growth factor 1 (IGF-1), upstream activator of PI3K in the heart, which is known to be responsible for exercise induced myocardial hypertrophy (10) and even for an increased cardiac contractility (11, 12). On the contrary, if PI3Kα signaling is attenuated, the numbers of L-type Ca channels on the cell surface decrease and thereby reduce *I*_Ca_ and cardiac contractility (13). Similarly, pharmacological inhibition of PI3K using LY-249002 prevents IGF-1 driven activation of intracellular Ca handling in adult rat cardiomyocytes, and adenoviral transfection of a dominant negative Akt prevents IGF-1 mediated upregulation of SERCA2a protein (14). These findings collectively show that both, increased transsarcolemmal Ca influx via the *I*_Ca_ as well as enhanced SR Ca loading (through PI3K/Akt-dependently upregulated SERCA2a) may contribute to activation of Ca handling upon IGF-1 exposure. Heart failure with impaired contractile function is due to a reduction in the amplitude of the systolic Ca transient as a result of decreased *I*_Ca_ through the L-type Ca channels (LTCC) as well as slowed SERCA2a leading to Ca-depletion of sarcoplasmic reticulum (SR) thus achieving less systolic Ca release (15).

In that regard, McMullen et al. provided interesting evidence that Ibrutinib is capable of inhibiting IGF-1 mediated signaling on the PI3K-Akt-pathway in neonatal rat ventricular myocytes (16). Accordingly, we tested the hypothesis that Ibrutinib might disturb IGF-1 mediated activation of intracellular Ca handling in adult ventricular cardiomyocytes.

## Methods

### Isolation and culture of mouse cardiomyocytes

The isolation and culture of ventricular cardiac myocytes was performed on adult (10–12 weeks) C57BL6/J wildtype mice (Charles River Laboratories, Wilmington/ USA). Murine hearts were carefully excised from isoflurane-anesthetized mice and retrogradely perfused with an isolation solution via Langendorff apparatus as described previously (17). After enzymatic digestion and mechanical dissociation of the heart, a gradual Ca reintroduction was performed. Isolated myocytes were plated on laminin-coated petri dishes in plating medium consisting of (in mmol/L): 2 L-Glutamine (biochrom AG), 10 2,3-Butanedione Monoxime (BDM, Thermo Scientific), 1% v/v Penicillin-Streptomycin (Sigma Aldrich), 1% v/v Insulin-Transferrin-Selenium-Sodium Pyruvate (gibco), 0.1% Bovine serum albumin (Thermo Scientific) dissolved in Minimum Essential Medium (MEM, Thermo Scientific) (18). IGF-1 (Sigma Aldrich) at 10 nmol/L was added to the experimental groups with or without Ibrutinib (AdooQ® Bioscience) at 1 µmol/L or Acalabrutinib at 10 µmol/L (MedChem Express). Groups without Ibrutinib and IGF-1 served as vehicle treated controls. Some cells were exposed to Ibrutinib at 1 µmol/L only. Cells were cultured for 24 ± 2 h at 37°C (5% O_2_ and 95% CO_2_). In order to analyze cell viability after treatment and to ensure cell survival, images of three fields of view per cell and culture chamber were recorded. Per field of view vital and avital cells were counted, respectively. Discrimination of cell viability was measured by morphological criteria, apoptotic cell rounding and shrinkage, and appearance of membrane bubbles. All experiments were performed in accordance with the Helsinki Declaration and with approval from the local authorities.

### Assessment of intracellular Ca handling properties using epifluorescence

**Figure 1:**
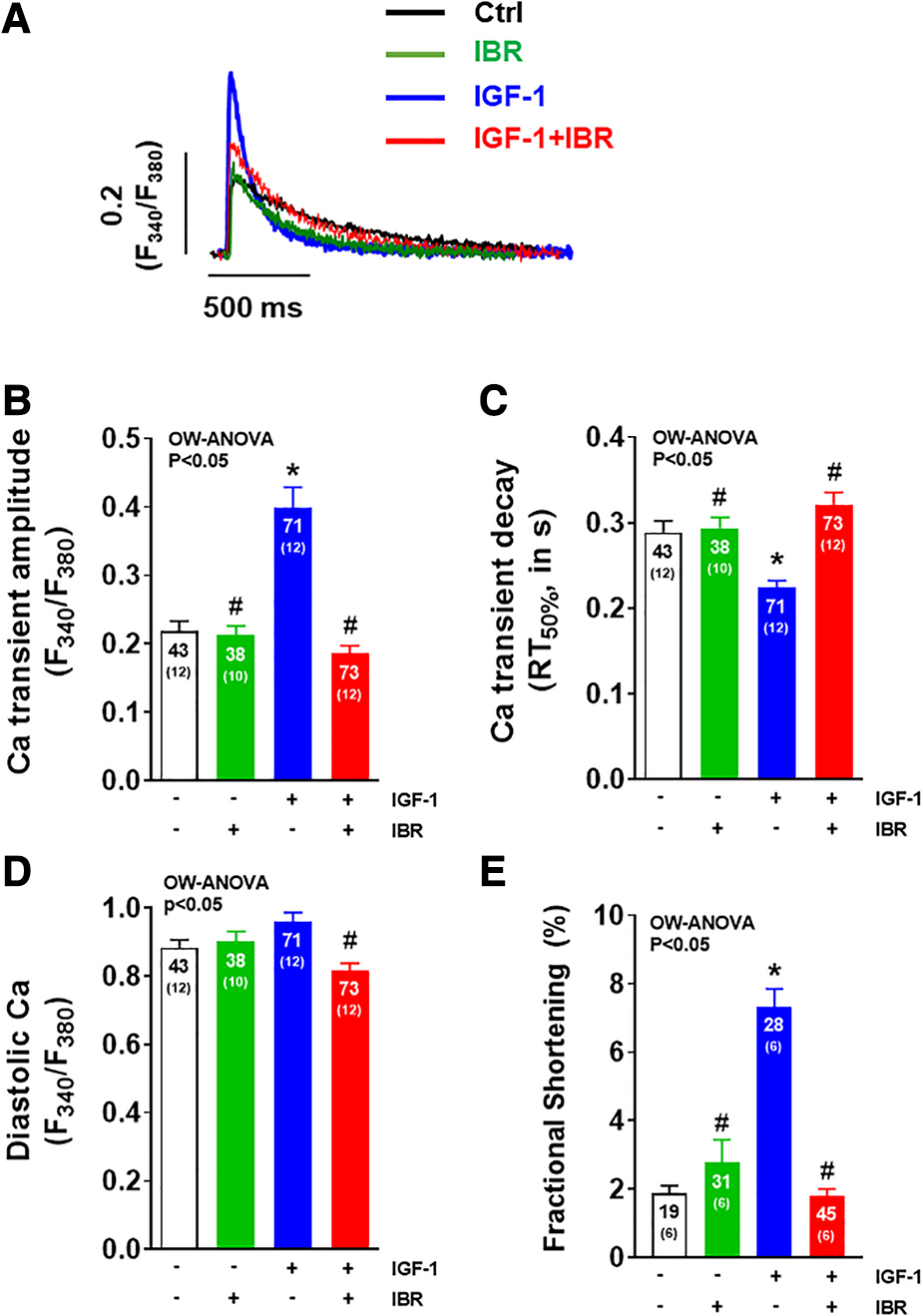
Ibrutinib impairs IGF-1-dependent activation of intracellular Ca handling. (**A**) Original traces of intracellular Ca transients measured in Fura-2 AM loaded ventricular myocytes following cell culture for 24 ± 2 h. (**B**) Mean data of Ca transient amplitudes showing that IGF-1 treatment increases Ca transient amplitudes by ∼83%. Ca transient amplitudes were significantly reduced in IBR ± IGF-1 treated myocytes as compared to myocytes treated solely with IGF-1. (**C**) Mean data for Ca transient decay (RT_50%_) as an approximation of SR Ca reuptake. IGF-1 is associated with a ∼22% acceleration of Ca transient decay as compared to untreated control cells, which was inhibited in myocytes treated with IBR ± IGF-1. (**D**) Mean data for diastolic Ca indicating significant decrease in cells treated with IBR + IGF-1 vs. IGF-1 alone. (**E**) Mean data for fractional shortening (% baseline sarcomere length) depict a significant increase in cells treated solely with IBF-1, whereas this effect was blocked by IBR ± IGF-1. *indicates significance vs. Ctrl, ^#^ indicates significance vs. IGF-1 using one-way ANOVA with Tukey's post-hoc test.

In order to investigate intracellular Ca handling, cultured myocytes firstly needed to be harvested, using a cell scraper, and subsequently plated on laminin-coated chambers, settling for 20 min. Chambers were then loaded with Fura-2 AM (Invitrogen) at 10 µmol/L in the presence of 0.02% (w/v) pluronic acid (Molecular Probes, Eugene, OR) and incubated for 15 min, at room temperature in darkness. The chambers were mounted on the stage of an inverted microscope (Nikon Eclipse TE2000-U) and superfused with experimental solution containing (in mmol/L) 140 NaCl, 4 KCl, 1 MgCl_2_, 5 HEPES, 10 glucose, 1 CaCl_2_, at 37°C (pH 7.4). Cells were electrical field-stimulated (voltage 25% above threshold) at 0.5 Hz for ∼5 min until reaching steady state conditions. Intracellular Fura-2 AM was excited at 340 nm and 380 nm (F_340_ and F_380_) and emitted fluorescence was detected at 510 nm. Ca transient amplitudes were calculated by the fluorescence ratio of F_340_/F_380_ after subtracting the background fluorescence at each excitation wavelength. Measurements were recorded and analyzed by IonWizard software (IonOptix Corporation, Boston, MA). Please note that data presented in Figures 1, 5 represent independent sets of experiments. Sarcoplasmic reticulum (SR) Ca content was evaluated by Ca transient amplitude after rapid application of caffeine (10 mmol/L, Sigma Aldrich) during pause of field stimulation. The Na/Ca-exchanger (NCX)-function was assessed by calculating the monoexponential time-constant *τ* (“tau”) of caffeine-induced Ca transient. Sarcomere length of cardiomyocytes was recorded using the Sarcomere Length Detection System (MyoCam, IonOptix Corporation, Boston, MA). After averaging contractions for ∼10 beats, fractional shortening (% baseline sarcomere length) was analyzed using IonWizard 6.4.1.73.

### Assessment of Ca spark frequency using confocal microscopy

Cells were loaded with Fluo-4 AM at 10 µmol/L in the presence of 0.02% (w/v) pluronic acid (Molecular Probes, Eugene, OR) and incubated for 15 min at room temperature in darkness. Chambers were then mounted on a confocal laser scanning microscope (Zeiss LSM 700) to assess spontaneous diastolic Ca release events from the SR (Ca sparks). Myocytes were excited via an argon laser (at 488 nm) and emitted fluorescence was collected after passing a 505 nm long-pass emission filter. Cells were superfused with normal Tyrode's solution (see above) and stimulated at 0.5 Hz. Ca sparks were analyzed using Zeiss Zen 3.1 software while line scans (512 pixel of 0.07 μm size, 1,319 lines per second, 10,000 lines per scan, 488 nm excitation, 505–530 nm emission) were performed immediately after termination of electrical field stimulation. Ca sparks were detected and quantified using Sparkmaster with visual confirmation of sparks detected. Ca spark frequency (CaSpF) was calculated and normalized to scanned myocyte width and scanning interval.

### Patch-clamp experiments

Ruptured-patch whole-cell voltage-clamp was used to measure *I*_Ca_ (voltage-clamp configuration) in cultured ventricular myocytes that had been prepared as described above. Myocytes were mounted on the stage of an inverted microscope (Nikon Eclipse TE2000-U). For *I*_Ca_ measurements, microelectrodes (2–3 MΩ) were filled with (in mmol/L) 86 CsCl, 40 Cs-glutamate, 0.92 MgCl_2_, 5 Mg-ATP, 0.3 Li-GTP, 10 HEPES, 5 EGTA and 1.8 CaCl_2_ [free (Ca^2+^)_i_ 100 nmol/L] (pH 7.2, CsOH). The bath solution contained (in mmol/L) 140 NaCl, 4 CsCl, 1 MgCl_2_, 10 glucose, 10 HEPES, 1 CaCl_2_ (pH 7.4, CsOH). For *I*_Ca_, cardiomyocytes needed to achieve a seal greater than 1 giga-Ohm and access resistance below 8 MΩ. Signals were filtered with 2.9 and 10 kHz Bessel filters and recorded with an EPC10 amplifier (HEKA Elektronik). Recordings were started 2–3 min after rupture. All experiments were conducted at room temperature. *I*_Ca_ was analyzed by subtracting steady state current from peak *I*_Ca_ current. In a next step, the amplitude was normalized by the cell capacity.

### Protein expression and phosphorylation levels

For western blot analysis, only cultured cardiomyocytes were used. After denaturation (for 30 min at 37°C in 10% β-mercaptoethanol), proteins were separated on 5% (RyR2, pS2814-RyR2, pS2809-RyR2), 8% (Akt, pS473-Akt, pI3Kα, SERCA) or 11% (PLB, pS16-PLB, pT17-PLB) SDS-polyacrylamide gels, then transferred to a nitrocellulose membrane (or PDVF membrane) and incubated with following primary antibodies: mouse monoclonal anti-phospho-serine473-Akt (1:1,000, BD Biosciences), mouse monoclonal anti-Akt (1:250, BD Biosciences), mouse monoclonal anti-SERCA2a (1:20,000, Thermo Scientific), rabbit monoclonal anti-PI3Kα (1:500, Thermo Scientific), mouse monoclonal anti-PLB (1:1,000, Thermo Scientific), rabbit polyclonal anti-phospho-serine16-PLB (1:500, Badrilla), rabbit polyclonal anti-phospho-threonine17-PLB (1:3,000, Badrilla), rabbit polyclonal anti-RyR2 (1:10,000, Sigma), rabbit polyclonal anti-phospho-serine2809-RyR2 (1:500, Badrilla), rabbit polyclonal anti-phospho-serin2814-RyR2 (1:3,000, Badrilla), and mouse monoclonal anti-GAPDH (1:50,000, BIOTREND) at 4°C overnight. Secondary antibodies were HRP-conjugated donkey anti-rabbit and sheep anti-mouse IgG (1:10,000, GE Healthcare) that were incubated for 1 h at room temperature. For chemiluminescent detection, Immobilon™ Western Chemiluminescent HRP Substrate (Millipore) was used. Values were afterwards normalized to control.

### Statistical analysis

For all experiments, investigators were blinded with respect to the intervention substance used. Unless otherwise stated, data are given as mean ± standard error of mean (SEM). Statistical analyses were performed using one and two-way ANOVA with Tukey's post-hoc test where appropriate. Values *P* < 0.05 were considered statistically significant.

## Results

### Ibrutinib impairs IGF-1-dependent activation of intracellular Ca handling

As illustrated by the representative Ca transients shown in Figure 1A, IGF-1 treatment (at 10 nmol/L for 24 h) resulted in an activation of intracellular Ca handling in terms of a significant increase in Ca transient amplitudes by ∼83% (*P* < 0.05) as it has been reported previously (average data in Figure 1B) (14). This was associated with a ∼22% (*P* < 0.05) acceleration of Ca transient decay (RT_50%_) indicating enhanced SR Ca reuptake as compared to untreated control cells (Figure 1C). Concomitant treatment of adult ventricular cardiomyocytes with IGF-1 and Ibrutinib (IBR, at 1 µmol/L for 24 h) completely prevented this IGF-1-dependent activation of intracellular Ca handling. In detail, Ca transient amplitudes were significantly reduced with 0.19± 0.01 a.u. in IGF-1 + IBR treated myocytes (*n* = 73) as compared to a mean Ca transient amplitude of 0.40± 0.03 a.u. in myocytes that were solely treated with IGF-1 (*n* = 71; *P* < 0.05, Figure 1B). Likewise, a significantly slower Ca transient decay indicating non-accelerated SR Ca reuptake was observed in myocytes treated with IGF-1 + IBR as compared to IGF-1-treated cells (Figure 1C). Cells treated with solely Ibrutinib had unchanged Ca transient amplitudes and decay kinetics as compared to vehicle treated control cells indicating no gross change in intracellular Ca handling upon sole IBR treatment. Interestingly, while there was only a numeric increase in diastolic Ca in IGF-1 treated myocytes as compared to vehicle control cells, concomitant IBR treatment resulted in a significant reduction of diastolic Ca (from 0.96 ± 0.03 a.u. vs. 0.82 ± 0.02 a.u.; *P* < 0.05, Figure 1D). Activated intracellular Ca-handling coincided with increased cellular contractility (i.e., fractional shortening) suggesting a positive inotropic effect of IGF-1 that can be largely inhibited by Ibrutinib (Figure 1E).

### Ibrutinib abrogates the IGF-1-induced increase of SR Ca content and *I*_Ca_

**Figure 2:**
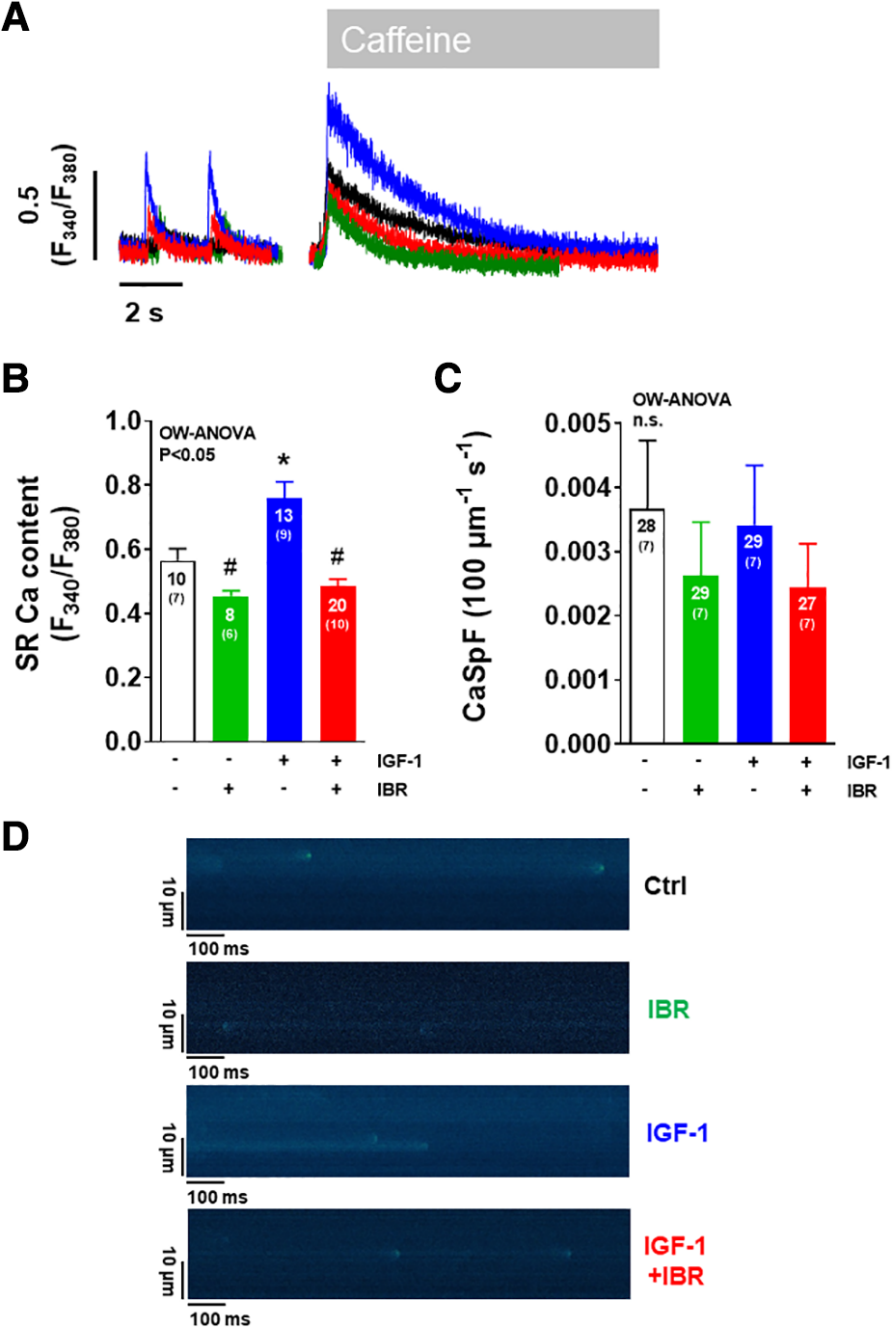
Ibrutinib abrogates the IGF-1-induced increase in SR Ca content. (**A**) Representative of caffeine-induced Ca transients illustrating increased SR Ca content in IGF-1 treated ventricular myocytes (blue). (**B**) Mean data for SR Ca content as assessed by caffeine-induced Ca transients portrays that SR Ca content was significantly increased by IGF-1. This effect was abrogated by concomitant treatment of cardiomyocytes with IGF-1 and Ibrutinib. (**C**) Mean data for diastolic Ca spark frequency (CaSpF) illustrate that diastolic Ca release from the SR as measured by Ca spark frequency was unaffected by either treatment modality. (**D**) Original confocal line scan images of isolated ventricular myocytes loaded with Fluo-4 AM. *indicates significance vs. Ctrl, ^#^ indicates significance vs. IGF-1 using one-way ANOVA with Tukey's post-hoc test.

**Figure 3:**
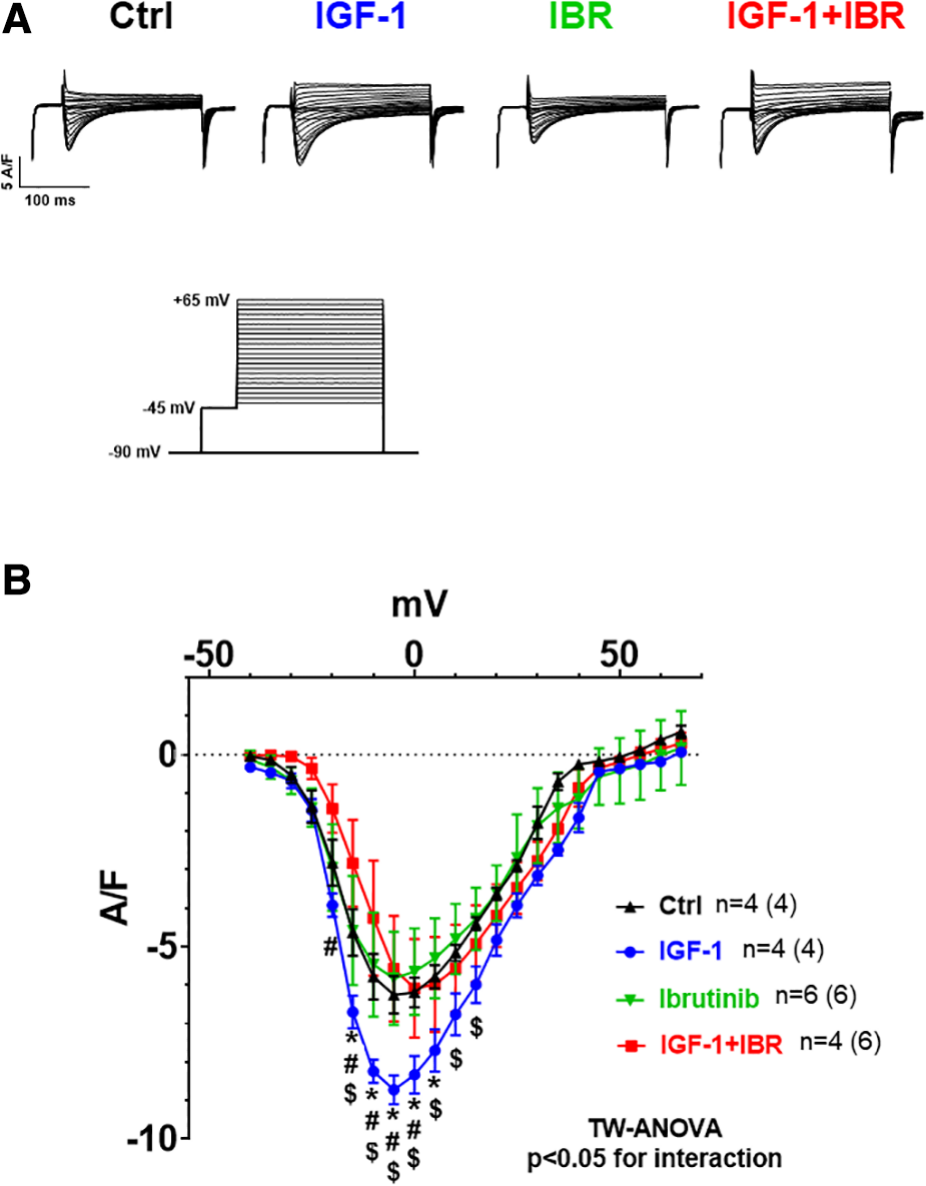
Ibrutinib abrogates the IGF-1-induced increase in *I*_Ca_. (**A**) Original traces of current-voltage relationship between different treatments (protocol depicted on the Fig. below 3A). (**B**) Mean data for peak *I*_Ca_-voltage relationship measured by whole cell patch clamp technique in isolated mouse ventricular myocytes revealed that IGF-1 significantly increased *I*_Ca_ as compared to untreated control cells, a phenomenon that was not present in myocytes that underwent combined IGF-1 + IBR treatment. *indicates significance vs. Ctrl, ^#^ indicates significance vs. IGF-1, ^$^ indicates significance vs. IBR using two-way ANOVA with Tukey's post-hoc test.

We next aimed to investigate the distinct functional mechanisms in intracellular Ca handling that were inhibited by Ibrutinib in IGF-1-treated ventricular myocytes. IGF-1 is known to activate Ca handling by increasing SR Ca load through upregulation of the PI3K-Akt-SERCA2a signaling cascade (14) and via stimulation of transarcolemmal Ca influx (*I*_Ca_) through the L-type Ca channels (LTCC) (19). In our model, IGF-1 significantly increased SR Ca content as well, from 0.56± 0.04 a.u. in untreated cells (*n* = 10) to 0.76± 0.05 a.u. in the presence of IGF-1 (*n* = 13, see representative caffeine-induced Ca transients in Figure 2A and mean data in Figure 2B). Importantly, as with Ca transient amplitudes, this increase was completely abrogated by concomitant treatment with IGF-1 and IBR in ventricular cardiomyocytes. Mechanistically, we interpret this finding as a result of Ibrutinib-dependent inhibition of SR Ca *loading* (compare Figure 1C), because diastolic Ca *release* from the SR as measured by Ca spark frequency was not affected by either IGF-1- or combined IGF-1 + IBR-treatment (Figures 2C,D). Transsarcolemmal Ca extrusion via the Na/Ca exchanger that can be approximated by the decay kinetics of the caffeine-induced Ca transient did not differ between groups (3,267± 865 ms in the presence of IGF-1, *n* = 12 vs. 2,315 ± 413 ms in the presence of IGF-1 + IBR, *n* = 17, *P* = 0.53). In a next step, transarcolemmal *I*_Ca_ was investigated. Original traces (Figure 3A) and quantitative data for the current-voltage relationship (Figure 3B) revealed that IGF-1 significantly increased *I*_Ca_ as compared to untreated control cells, a phenomenon that was not present in myocytes that underwent combined IGF-1 + IBR treatment. In detail, peak *I*_Ca_ was increased to 8.72 ± 0.37 A/F at −5 mV in IGF-1 treated myocytes (*n* = 4) as compared to 6.25 ± 0.49 A/F in control cells (*n* = 4), and to 5.58± 1.37 A/F in IGF-1 + IBR myocytes (*n* = 4), respectively.

### Ibrutinib attenuates Akt signaling and SERCA2a upregulation in IGF-1 treated ventricular myocytes

**Figure 4:**
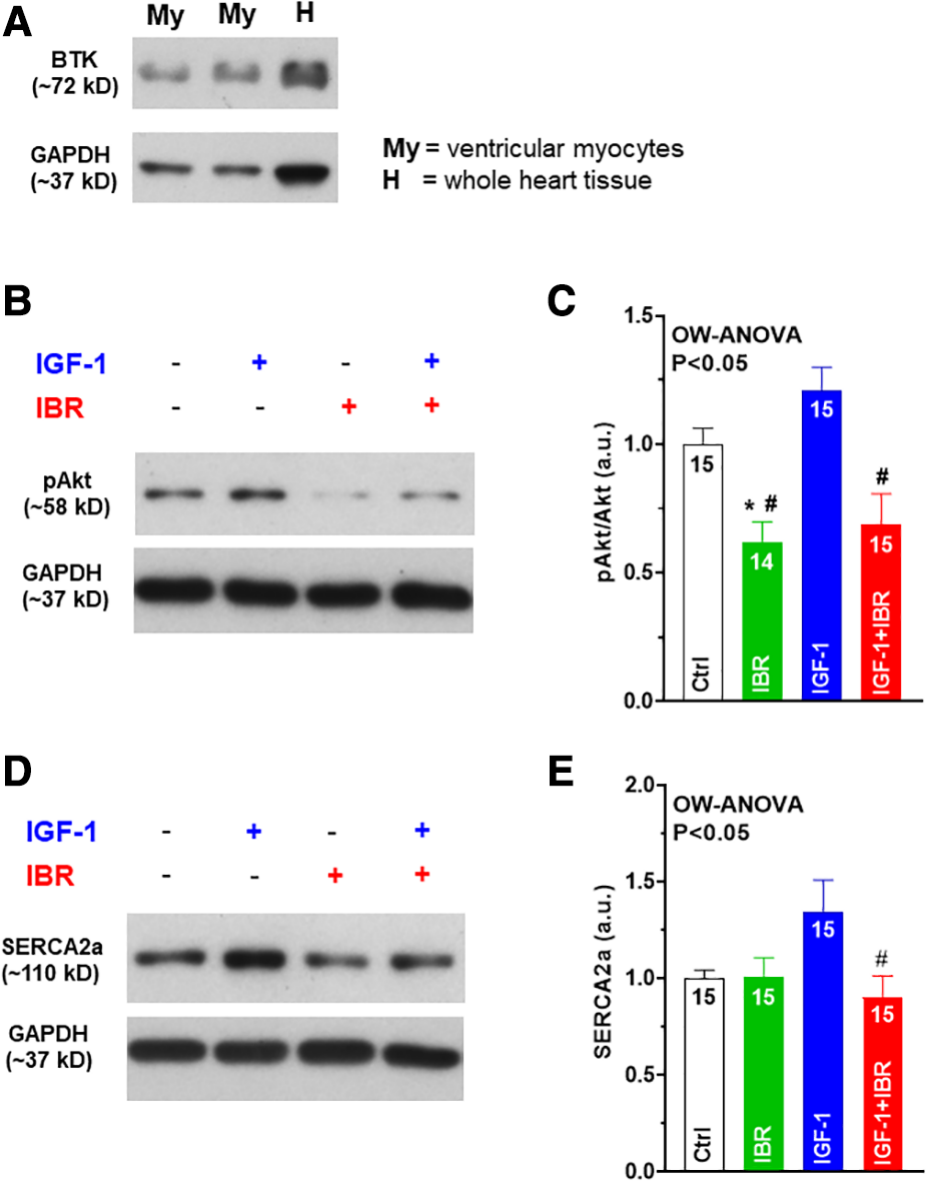
Ibrutinib attenuates Akt signaling and SERCA2a upregulation in IGF-1 treated ventricular myocytes. (**A**) Confirmation of robust cardiac expression levels of BTK in our murine experimental model (i.e. in isolated ventricular myocytes (My) as well as in whole heart tissue (**H**)). (**B**) Original western blots of phosphorylated Akt (at Ser473) in the presence or absence of IGF-1 or IBR. (**C**) Mean data of IGF-1-treated myocytes revealed significantly suppressed activation of Akt in the presence of IBR as indicated by the decreased ratio of phosphorylated Akt (at Ser473) to Akt expression. (**D**) Original western blots of SERCA2a in the presence or absence of IGF-1 or IBR. (**E**) Mean data of SERCA2a expression was found to be numerically increased following IGF-1 treatment, but significantly reduced in case of combined IGF-1 + IBR treatment. *indicates significance vs. Ctrl, ^#^ indicates significance vs. IGF-1 using one-way ANOVA with Tukey's post-hoc test.

Next, we aimed to investigate whether PI3K/Akt signaling might be affected by concomitant IGF-1 ± IBR treatment. At first, we confirmed robust cardiac expression levels of BTK in our murine experimental model (i.e., in isolated ventricular myocytes as well as in whole heart tissue, see Figure 4A). IGF-1-treated myocytes revealed significantly suppressed activation of Akt in the presence of IBR as indicated by the decreased ratio of phosphorylated Akt (at serin-473) to Akt expression (see original western blots in Figure 4B and mean data in Figure 4C and Table 1). As already mentioned, IGF-1-induced and PI3K/Akt-mediated expression of SERCA2a is a known activator of intracellular Ca handling in isolated cardiac myocytes (14). In line with that, we found SERCA2a expression to be numerically increased following IGF-1 treatment as well (to 1.35 + 0.16 a.u, *n* = 15), and to be significantly lower in case of combined IGF-1 + IBR treatment (0.90 + 0.11 a.u., *n* = 15; see original western blots in Figure 4D and mean data in Figure 4E and Table 1). Because increased phosphorylation of phospholamban (PLB) that inhibits SERCA2a activity in its unphosphorylated state could also have caused increased SR Ca loading upon IGF-1 treatment, we also assessed PLB phosphorylation status. However, we did not observe hyperphosphorylation of PLB at serine-16 (the PKA-specific phosphorylation site) nor at threonine-17 (the CaMKII-specific site) in IGF-1 treated myocytes (Table 1). Similarly, no alterations with respect to the phosphorylation status of the SR Ca release channels (i.e., the RyR2) were observed at serine 2809 (the PKA-specific site) nor at 2814 (the CaMKII-specific site) in the presence of IGF-1 in our model (Table 1).

**Table 1:** Expression and phosphorylation status of key cardiac Ca handling proteins.

Protein	Ctrl	*n*	IBR		IGF-1	*n*	IGF-1 + IBR	*n*
pS473/Akt	1.00 ± 0.06	15	0.62 ± 0.08^a,b^	14	1.21 ± 0.09	15	0.70 ± 0.12^b^	15
SERCA2a	1.00 ± 0.04	15	1.01 ± 0.10	15	1.35 ± 0.16	15	0.90 ± 0.11^b^	15
PI3Kα	1.00 ± 0.04	13	1.01 ± 0.14	13	1.00 ± 0.12	12	0.82 ± 0.08	13
PLB	1.00 ± 0.04	14	1.10 ± 0.20	14	1.33 ± 0.26	14	1.06 ± 0.20	14
pS16/PLB	1.00 ± 0.14	12	1.08 ± 0.30	12	1.19 ± 0.30	12	1.10 ± 0.24	12
pT17/PLB	1.00 ± 0.04	14	0.97 ± 0.09	14	0.86 ± 0.08	14	1.01 ± 0.08	14
RyR2	1.00 ± 0.08	10	0.94 ± 0.07	10	1.06 ± 0.11	10	1.01 ± 0.10	10
pS2809/RyR2	1.00 ± 0.15	10	0.78 ± 0.11	10	1.00 ± 0.14	10	1.02 ± 0.12	10
pS2814/RyR2	1.00 ± 0.09	9	0.93 ± 0.17	9	1.02 ± 0.15	9	0.94 ± 0.12	9

pS473, Phosphorylation at 473 (Akt); Akt, Protein kinase B; SERCA2a, sarcoplasmic reticulum Ca-ATPase; PI3Kα, phosphoinositide 3-kinase alpha; PLB, Phospholamban; pS16, Phosphorylation at Serine 16 (PLB); pT17, Phosphorylation at Threonine 17 (PLB); RyR2, Ryanodine Receptor 2; pS2809, Phosphorylation at Serine 2809 (RyR2); pS2814, Phosphorylation at Serine 2814 (RyR2).

^a^ Indicates significance vs. Ctrl.

^b^ Indicates significance vs. IGF-1 using Tukey's post-hoc test following one-way ANOVA.

### The second generation BTK inhibitor Acalabrutinib impairs IGF-1-dependent activation of intracellular Ca handling

**Figure 5:**
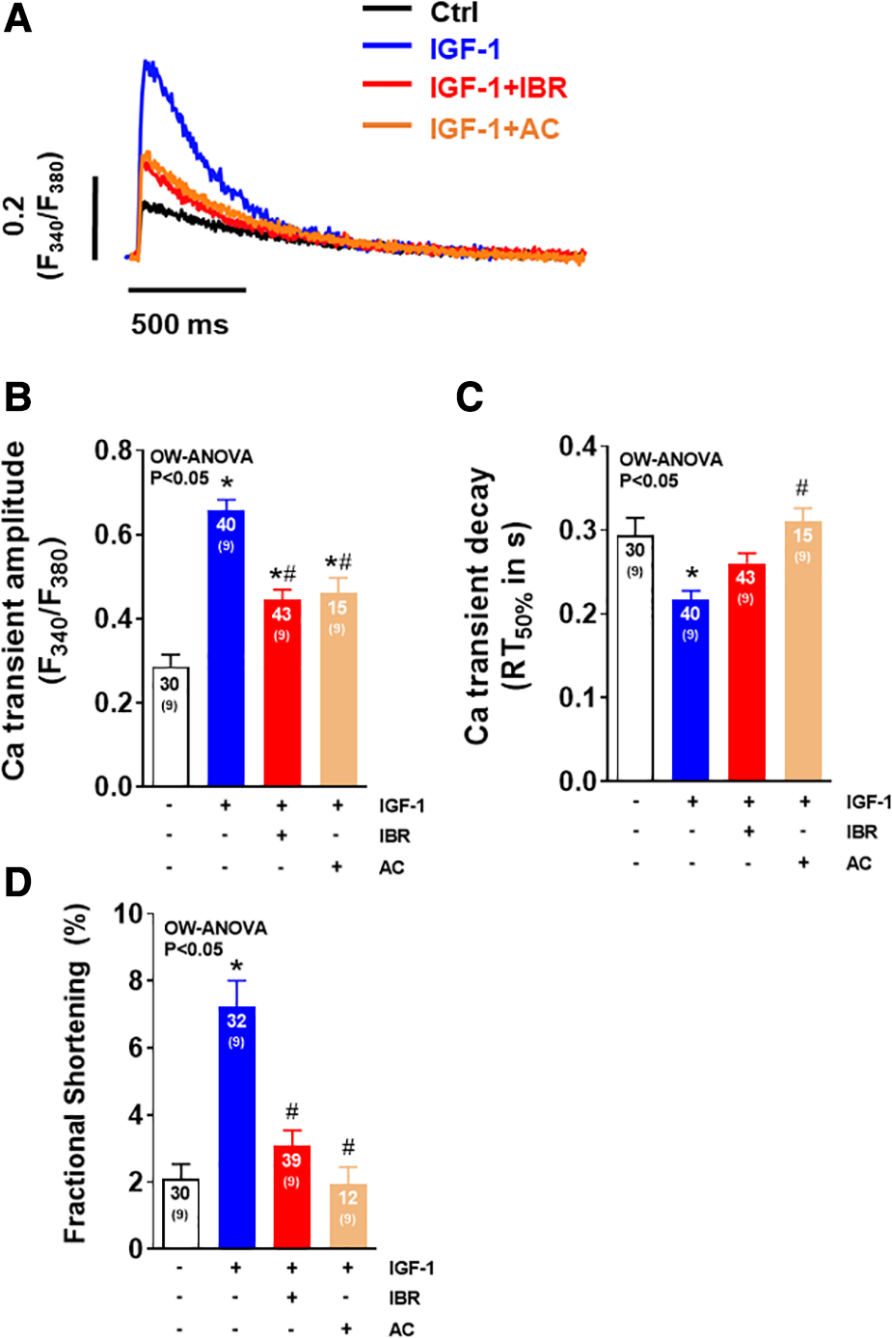
The second generation BTK inhibitor acalabrutinib impairs IGF-1-dependent activation of intracellular Ca handling. (**A**) Original traces of intracellular Ca transients measured in Fura-2 AM loaded ventricular myocytes in cell culture 24 ± 2 h. (**B**) Mean data of Ca transient amplitudes showing that IGF-1 significantly increased Ca transient amplitudes by 230%, an effect that was comparably blocked by Ibrutinib and Acalabrutinib (AC). (**C**) Mean data of SR Ca reuptake as approximated by Ca transient decay (RT_50%_). Ibrutinib and Acalabrutinib (AC) both slowed Ca transient decay compared to IGF-1 treated myocytes. (**D**) Mean data for fractional shortening (% baseline sarcomere length) show a significant increase in cells treated solely with IGF-1, whereas this effect was blocked by IBR + IGF-1 and by AC + IGF-1. *indicates significance vs. Ctrl, ^#^ indicates significance vs. IGF-1 using one-way ANOVA with Tukey's post-hoc test.

In a final step, we tested whether the second generation BTK inhibitor Acalabrutinib (AC) with greater BTK selectivity and lower cardiovascular toxicity (20) would also impair IGF-1-mediated activation of intracellular Ca handling. As illustrated by the representative Ca transients in Figure 5A, IGF-1 again significantly increased Ca transient amplitudes in vehicle treated cells, an effect that was comparably reduced by both Ibrutinib and Acalabrutinib (Figure 5B). However, in this independent set of data, in which IGF-1 increased Ca transient amplitude even by almost ∼230%, neither IBR nor AC were able to fully antagonize the effect of IGF-1 on Ca transients (as shown in Figure 1), which suggests that there might a dose-dependent effect of pharmacological BTK-inhibition with respect to IGF-1 regulated Ca handling. Nevertheless, both drugs slowed Ca transient decay (Figure 5C), which points to a functional class effect of BTK inhibitors in cardiac myocytes. Supporting this conclusion, cells treated with IGF-1 revealed a significant increase in fractional shortening as compared to untreated cells, which was largely prevented in cells treated with both IBR + IGF-1 or AC + IGF-1 (Figure 5D). In order to follow up a potential dose-dependency of Ibrutinib on IGF-1 dependent Ca handling, cells were exposed to IGF-1 (at 10 nmol/L) and to Ibrutinib at increasing concentrations of IBR (i.e., at 0.01 µM, 0.1 µM, 1 µM, and 2 µM). Supplementary Figure S1 shows that Ibrutinib is capable of largely hampering IGF-1 dependent activation of intracellular Ca handling at a concentration of 0.1 µM with more potent inhibition with increasing concentrations of IBR that suggests a dose-dependent effect of BTK-inhibition on IGF-1 signaling. Importantly, cell survival upon culture was not different in between groups (Supplementary Figure S2).

## Discussion

Our study is the first to our knowledge that reveals potential pathomechanisms of the BTK-inhibitor Ibrutinib in murine *ventricular* cardiomyocytes. Our findings coincide with recent real-world data from VigiBase, the World Health Organization's (WHO) global database of individual case safety reports, portraying those patients treated with Ibrutinib are at higher risk of developing heart failure (5). Mechanistically, our study implies that cultured adult mouse ventricular myocytes lack IGF-1-related activation of intracellular Ca handling in a dose-dependent manner when concomitantly treated with Ibrutinib. Ibrutinib inhibits IGF-1 dependent Akt phosphorylation, as well as the resulting positive inotropic effect in ventricular myocytes. In contrast, we did not observe dysfunctional Ca handling in Ibrutinib treated cardiomyocytes in the absence of IGF-1 (despite relevant hypophosphorylation of Akt), which suggests that IGF-1 is needed to uncover the detrimental role of BTK-inhibition in ventricular cardiomyocytes. Consequently, Ibrutinib-dependent inhibition of IGF-1/PI3K/Akt signaling in ventricular myocytes may contribute to left ventricular dysfunction as observed in patients under treatment with Ibrutinib.

IGF-1/PI3K/Akt signaling pathway is a key mediator in regulating adaptive exercise-induced cardiac hypertrophy, thus inheriting the pivotal role of preserving ventricular myocyte function. The cardiac protection is triggered by a gain- and loss-of-function of the IGF-1/PI3K/Akt signaling, proven in numerous studies with genetically modified mouse models. Mice with compromised IGF-1/PI3K/Akt signaling are shown to be more susceptible to pathological remodeling (21). On the other hand, Matsui et al. showed that neonatal rat cardiomyocytes are protected from hypoxia-induced apoptosis by stimulation of the IGF-1/Akt signaling pathway (22). During the 90′s first studies showed that the upstream effector, IGF-1, exerts a positive inotropic effect on cardiomyocytes (23, 24) thereby maintaining cardiac contractility in different stress conditions. Even failing human myocardium was observed to have an IGF-1 induced Ca-dependent positive inotropic effect (25). Enhanced contractile function due to IGF-1 exposure was seen to be a direct consequence of an increase in the amplitude of the systolic Ca transient, which results from increased transarcolemmal Ca influx (*I*_Ca_) through LTCC, as well as an accelerated SR Ca reuptake, achieving higher SR Ca deposit and thereby increasing systolic Ca release (26). Similar results were shown by Sun et al. displaying an IGF-1 induced increase of *I*_Ca_ through PKB/Akt dependent activation of LTCC in murine ventricular myocytes (19). Unfortunately, the IGF-1/PI3K/Akt signaling pathway also plays a critical role in tumor cell survival and proliferation, imposing a potential conflict when cancer therapy is imminent and physicians try to unravel tumor suppression and prevention of cardiotoxicity. BTK-inhibition targets tumor apoptosis by disturbing PI3K/Akt signaling. In that regard, McMullen et al. were the first to show that the BTK-inhibitor, Ibrutinib, inhibits PI3K and Akt, in neonatal rat myocytes, even under stimulation with the upstream effector IGF-1. However, the pathophysiological effects of Ibrutinib on the heart are not entirely understood and just little is known.

Up until now, particularly atrial cells were examined, since atrial fibrillation is the clinically most striking adverse event during therapy with Ibrutinib. Chemoproteomic profiling identified C-terminal Src kinase (csk) as being the most durable candidate for Ibrutinib-induced AF. Experiments with csk knockout mice portrayed that csk inhibition led to increased AF, presenting a potential pathomechanism for Ibrutinib-induced AF without further exploring cellular pathomechanisms (27). One well accepted cellular pathomechanism of AF is a decreased SR Ca content as a consequence of enhanced diastolic SR Ca loss. In that regard, the stress kinase Ca/calmodulin-dependent protein kinase II (CaMKII) has been elaborated to play a vital role for SR Ca leakage in atrial fibrillation. Moreover, activated CaMKII and resulting SR Ca leakage are hallmarks of failing myocytes. It has been shown that enhanced phosphorylation of the Ryanodin receptor 2 (RyR2), induced by CaMKII, leads to an increase of SR Ca leak in human myocardium during AF (28) and HF (29). Interestingly, CaMKII-dependent SR Ca leak is also observed in murine cells during AF undergoing Ibrutinib therapy (30), which suggests that increased CaMKII-activity could be relevant in our model as well. However, we did not observe increased phosphorylation of CaMKII or its specific-binding-sites at Thr17-PLB or Ser2814-RyR2, nor was there a significant difference of diastolic Ca leak between different treatments in our model. Nonetheless, while no changes were seen *acutely* (i.e., after 24 h of cell culture), this might change over time with a chronic approach. It remains unclear whether and to what extent a failure of IGF-1-dependent signaling ultimately favors CaMKII-dependent effects in Ibrutinib-treated myocytes. On the other hand, our data clearly show that Ibrutinib is capable of inhibiting two central mechanisms in ventricular cardiomyocytes through which IGF-1 exerts its positive inotropic potential, namely increased *I*_Ca_, and enhanced SR Ca loading, most likely as a consequence of reduced Akt signaling. Increased SR Ca loading in IGF-1 treated ventricular cardiac myocytes was associated with phosphorylated Akt and upregulated SERCA2a expression, but was not due to hyperphosphorylation of PLB at Thr17 or Ser16 (which would enhance SR Ca loading) nor with hypophosphorylation of RyR2 at Ser2808 or Ser2814 (which would result in reduced SR Ca loss), declaring specific protein expression regulation. In line with this, no spontaneous diastolic Ca release was observed, additionally arguing against an acute kinase-mediated effect, and corroborating the importance of upregulated SERCA2a function for elevated SR Ca loading upon IGF-1 exposure. Secondly, IGF-1 treated cells revealed an increased *I*_Ca_ as previously reported (19), a phenomenon that was completely absent when cells were concomitantly treated with Ibrutinib. Taken together, these observations collectively show that Ibrutinib is capable of inhibiting IGF-1 mediated activation of intracellular Ca handling by preventing two well accepted IGF-1 dependent effects on cardiac Ca handling that is SERCA2a upregulation and stimulation of *I*_Ca_ (14, 19). In terms of cell survival, no acute difference was found between treatment groups which argues against higher risk of cell death during Ibrutinib treatment in our model as a consequence of inhibited IGF-1 signaling as it has been previously observed in other cell lines (31). Finally, in order to obtain data regarding a potential off-target effect of Ibrutinib we also analyzed the effects on Ca handling of another more selective BTK inhibitor, Acalabrutinib, which has been ascribed a more favorable cardiac toxicity profile than Ibrutinib. Besides our finding showing that Acalabrutinib exhibits similar effects on Ca handling as Ibrutinib, Acalabrutinib was just recently described as being associated with ventricular arrhythmias after therapy initiation (32), suggesting a functional class effect of these BTK inhibitors with respect to ventricular dysfunction which would be in contrast to the observation by Xiao et al. that Acalabrutinib is not relevant in terms of Ibrutinib/csk-induced AF (27).

## Limitations and potential clinical implications

Our study has several important limitations that need to be considered. First, murine cells have different ion channel properties, consequently translation to human pathology should be regarded with caution. Experiments were performed solely in-vitro and more or less acutely (24 h cell culture). Although, the results concluding from our experiments may not reflect on a long-term *in-vivo* effect at organ level, this study provides first evidence for a potential cellular mechanism of Ibrutinib in ventricular cells. In order to achieve a more in-depth knowledge in-vivo experiments are eligible. This study cannot prove that Ibrutinib is capable of completely inhibiting IGF-1-dependent signaling in ventricular cardiomyocytes. We observed a considerably different extent of the inhibitory effect of IBR on IGF-1 regulated Ca handling in two independent sets of experiments (see Figures 1, 5), which we believe is due to the fact that independent experimental series can be associated with different quality of cell isolation and an accordingly different behavior of cells upon cell culture. Still, we believe that our study provides strong evidence for a dose-dependent reduction of IGF-1 signaling by pharmacological BTK-inhibition.

According to the current cardio-oncology guidelines of the European Society of Cardiology, patients who are treated with Ibrutinib should be monitored for left ventricular function (33). In the absence of an adaptive hypertrophic trigger, patients may remain asymptomatic upon clinical and echocardiographic assessment, which would fit to our observation that cells treated with Ibrutinib only revealed no apparent alteration in intracellular Ca handling. However, in case of a relevant adaptive hypertrophic context (i.e., intensified physical activity or even pregnancy) these patients may become clinically apparent and should rather be more closely monitored because dysfunctional IGF-1 signaling may be of particular relevance here. Further preclinical studies are required to understand the possible effects of Ibrutinib on the development of HF and thereby creating awareness for potential risk factors for physicians.

## Data Availability

The raw data supporting the conclusions of this article will be made available by the authors, without undue reservation.

## References

[1] Byrd JC Brown JR O’Brien S Barrientos JC Kay NE Reddy NM Ibrutinib versus ofatumumab in previously treated chronic lymphoid leukemia. N Engl J Med. (2014) 371(3):213–23. 10.1056/NEJMoa1400376 24881631 PMC4134521

[2] Byrd JC Furman RR Coutre SE Flinn IW Burger JA Blum KA Targeting BTK with ibrutinib in relapsed chronic lymphocytic leukemia. N Engl J Med. (2013) 369(1):32–42. 10.1056/NEJMoa1215637 23782158 PMC3772525

[3] Wang ML Rule S Martin P Goy A Auer R Kahl BS Targeting BTK with ibrutinib in relapsed or refractory mantle-cell lymphoma. N Engl J Med. (2013) 369(6):507–16. 10.1056/NEJMoa1306220 23782157 PMC4513941

[4] Castillo JJ Palomba ML Advani R Treon SP. Ibrutinib in Waldenström macroglobulinemia: latest evidence and clinical experience. Ther Adv Hematol. (2016) 7(4):179–86. 10.1177/2040620716654102 27493708 PMC4959643

[5] Salem JE Manouchehri A Bretagne M Lebrun-Vignes B Groarke JD Johnson DB Cardiovascular toxicities associated with ibrutinib. J Am Coll Cardiol. (2019) 74(13):1667–78. 10.1016/j.jacc.2019.07.056 31558250

[6] Ghigo A Laffargue M Li M Hirsch E. PI3K and calcium signaling in cardiovascular disease. Circ Res. (2017) 121(3):282–92. 10.1161/CIRCRESAHA.117.310183 28729453

[7] Craxton A Jiang A Kurosaki T Clark EA. Syk and Bruton’s tyrosine kinase are required for B cell antigen receptor-mediated activation of the kinase Akt. J Biol Chem. (1999) 274(43):30644–50. 10.1074/jbc.274.43.30644 10521450

[8] McMullen JR Amirahmadi F Woodcock EA Schinke-Braun M Bouwman RD Hewitt KA Protective effects of exercise and phosphoinositide 3-kinase(p110alpha) signaling in dilated and hypertrophic cardiomyopathy. Proc Natl Acad Sci U S A. (2007) 104(2):612–7. 10.1073/pnas.0606663104 17202264 PMC1766433

[9] Weeks KL Gao X Du XJ Boey EJH Matsumoto A Bernardo BC Phosphoinositide 3-kinase p110α is a master regulator of exercise-induced cardioprotection and PI3K gene therapy rescues cardiac dysfunction. Circ Hear Fail. (2012) 5(4):523–34. 10.1161/CIRCHEARTFAILURE.112.966622 22705768

[10] Neri Serneri GG Boddi M Modesti PA Cecioni I Coppo M Padeletti L Increased cardiac sympathetic activity and insulin-like growth factor-I formation are associated with physiological hypertrophy in athletes. Circ Res. (2001) 89(11):977–82. 10.1161/hh2301.100982 11717153

[11] Ghigo A Li M. Phosphoinositide 3-kinase: friend and foe in cardiovascular disease. Front Pharmacol. (2015) 6:1–7. 10.3389/fphar.2015.00169 26321955 PMC4534856

[12] Yano N Tseng A Zhao TC Robbins J Padbury JF Tseng Y-T. Temporally controlled overexpression of cardiac-specific PI3Kα induces enhanced myocardial contractility—a new transgenic model. Am J Physiol Circ Physiol. (2008) 295(4):H1690–4. 10.1152/ajpheart.00531.2008 PMC2593510 18723766

[13] Lu Z Jiang Y-P Wang W Xu X-H Mathias RT Entcheva E Loss of cardiac phosphoinositide 3-kinase p110 results in contractile dysfunction. Circulation. (2009) 120(4):318–25. 10.1161/CIRCULATIONAHA.109.873380 19597047 PMC2734096

[14] Kim S-J Abdellatif M Koul S Crystal GJ. Chronic treatment with insulin-like growth factor I enhances myocyte contraction by upregulation of Akt-SERCA2a signaling pathway. AJP Hear Circ Physiol. (2008) 295(1):H130–5. 10.1152/ajpheart.00298.2008 PMC2494754 18456736

[15] Bers DM Despa S. Cardiac excitation–contraction coupling. In: Encyclopedia of biological chemistry. Amsterdam, Netherland: Elsevier (2013). p. 379–83. Available from: http://linkinghub.elsevier.com/retrieve/pii/B9780123786302002218

[16] McMullen JR Boey EJH. Correspondence to the editor: ibrutinib increases the risk of atrial fibrillation, potentially through inhibition of cardiac. Blood. (2014) 124(25):3829–31. 10.1182/blood-2014-10-604272 25498454

[17] Sag CM Köhler AC Anderson ME Backs J Maier LS. CaMKII-dependent SR Ca leak contributes to doxorubicin-induced impaired Ca handling in isolated cardiac myocytes. J Mol Cell Cardiol. (2011) 51(5):749–59. 10.1016/j.yjmcc.2011.07.016 21819992 PMC3226826

[18] Hofhuis J Bersch K Büssenschütt R Drzymalski M Liebetanz D Nikolaev VO Dysferlin mediates membrane tubulation and links T-tubule biogenesis to muscular dystrophy. J Cell Sci. (2017) 130(5):841–52. 10.1242/jcs.198861 28104817

[19] Sun H Kerfant BG Zhao D Trivieri MG Oudit GY Penninger JM Insulin-like growth factor-1 and PTEN deletion enhance cardiac L-type Ca 2 + currents via increased PI3Kα/PKB signaling. Circ Res. (2006) 98(11):1390–7. 10.1161/01.RES.0000223321.34482.8c 16627784

[20] Byrd JC Hillmen P Ghia P Kater AP Chanan-Khan A Furman RR Acalabrutinib versus ibrutinib in previously treated chronic lymphocytic leukemia: results of the first randomized phase III trial. J Clin Oncol. (2021) 39(31):3441–52. 10.1200/JCO.21.01210 34310172 PMC8547923

[21] Sadasivan C Zhabyeyev P Labib D White JA Paterson DI Oudit GY. Cardiovascular toxicity of PI3Kα inhibitors. Clin Sci. (2020) 134(19):2595–622. 10.1042/CS20200302 33063821

[22] Matsui T Li L del Monte F Fukui Y Franke TF Hajjar RJ Adenoviral gene transfer of activated phosphatidylinositol 3′-kinase and akt inhibits apoptosis of hypoxic cardiomyocytes in vitro. Circulation. (1999) 100(23):2373–9. 10.1161/01.CIR.100.23.2373 10587343

[23] Freestone NS Ribaric S Mason WT. The effect of insulin-like growth factor-1 on adult rat cardiac contractility. Mol Cell Biochem. (1996) 163–164:223–9. 10.1007/BF00408662 8974061

[24] Kinugawa S. Positive inotropic effect of insulin-like growth factor-1 on normal and failing cardiac myocytes. Cardiovasc Res. (1999) 43(1):157–64. 10.1016/S0008-6363(99)00058-9 10536700

[25] Von Lewinski D Voß K Hülsmann S Kögler H Pieske B. Insulin-like growth factor-1 exerts Ca2+-dependent positive inotropic effects in failing human myocardium. Circ Res. (2003) 92(2):169–76. 10.1161/01.RES.0000051885.70159.12 12574144

[26] Aulbach F Simm A Maier S Langenfeld H Walter U Kersting U Insulin stimulates the L-type Ca2 + current in rat cardiac myocytes. Cardiovasc Res. (1999) 42(1):113–20. 10.1016/S0008-6363(98)00307-1 10435002

[27] Xiao L Salem JE Clauss S Hanley A Bapat A Hulsmans M Ibrutinib-mediated atrial fibrillation attributable to inhibition of C-terminal Src kinase. Circulation. (2020) 142(25):2443–55. 10.1161/CIRCULATIONAHA.120.049210 33092403 PMC9661397

[28] Neef S Dybkova N Sossalla S Ort KR Fluschnik N Neumann K CaMKII-Dependent diastolic SR Ca2 + leak and elevated diastolic Ca2 + levels in right atrial myocardium of patients with atrial fibrillation. Circ Res. (2010) 106(6):1134–44. 10.1161/CIRCRESAHA.109.203836 20056922

[29] Fischer TH Herting J Tirilomis T Renner A Neef S Toischer K Ca2+/calmodulin-dependent protein kinase ii and protein kinase a differentially regulate sarcoplasmic reticulum Ca2 + leak in human cardiac pathology. Circulation. (2013) 128(9):970–81. 10.1161/CIRCULATIONAHA.113.001746 23877259

[30] Jiang L Li L Ruan Y Zuo S Wu X Zhao Q Ibrutinib promotes atrial fibrillation by inducing structural remodeling and calcium dysregulation in the atrium. Hear Rhythm. (2019) 16(9):1374–82. 10.1016/j.hrthm.2019.04.008 30959203

[31] Tagoug I de Chalon AS Dumontet C. Inhibition of IGF-1 signalling enhances the apoptotic effect of AS602868, an IKK2 inhibitor, in multiple myeloma cell lines. PLoS One. (2011) 6(7):e22641. 10.1371/journal.pone.0022641 21799925 PMC3143180

[32] Bhat SA Gambril J Azali L Chen ST Rosen L Palettas M Ventricular arrhythmias and sudden death events following acalabrutinib initiation. Blood. (2022) 140(20):2142–5. 10.1182/blood.2022016953 35917449 PMC10405526

[33] Lyon AR López-Fernández T Couch LS Asteggiano R Aznar MC Bergler-Klein J 2022 ESC guidelines on cardio-oncology developed in collaboration with the European hematology association (EHA), the European society for therapeutic radiology and oncology (ESTRO) and the international cardio-oncology society (IC-OS). Eur Heart J. (2022) 43(41):4229–361. 10.1093/eurheartj/ehac244 36017568

