# Supplementary figures and images for: Ibrutinib impairs IGF-1-dependent activation of intracellular Ca handling in isolated mouse ventricular myocytes
Source: Front Cardiovasc Med. 2023 Aug 15;10:1190099. doi: 10.3389/fcvm.2023.1190099 (PMC10466040; doi:10.3389/fcvm.2023.1190099)

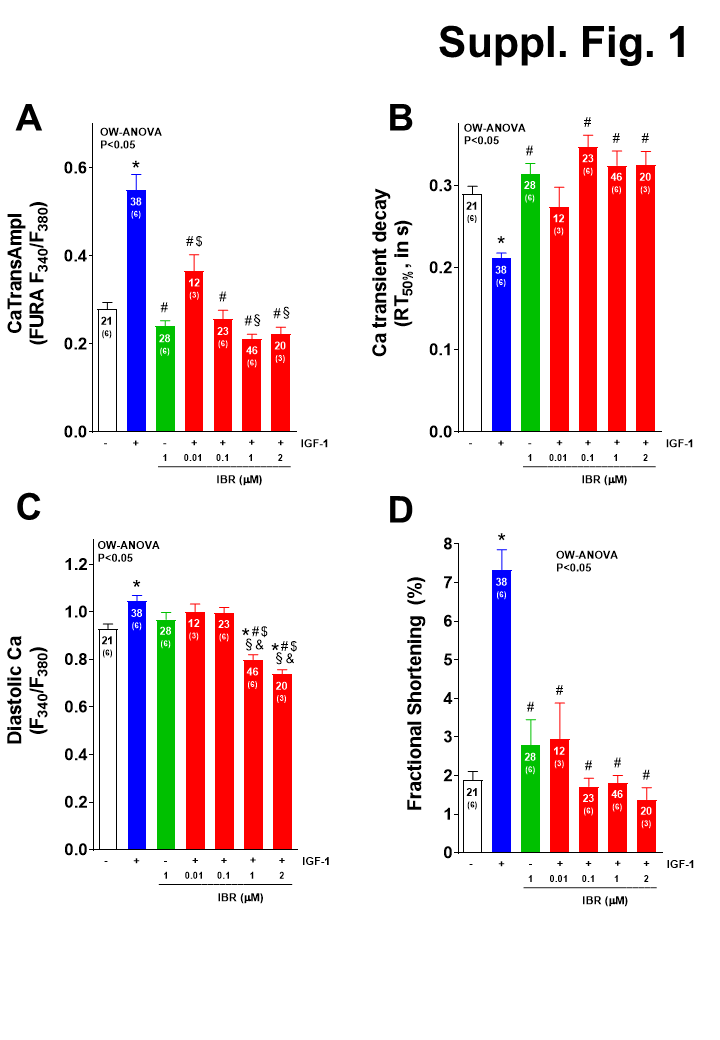

Supplement: Supplementary file 1 [file Image1.tif]

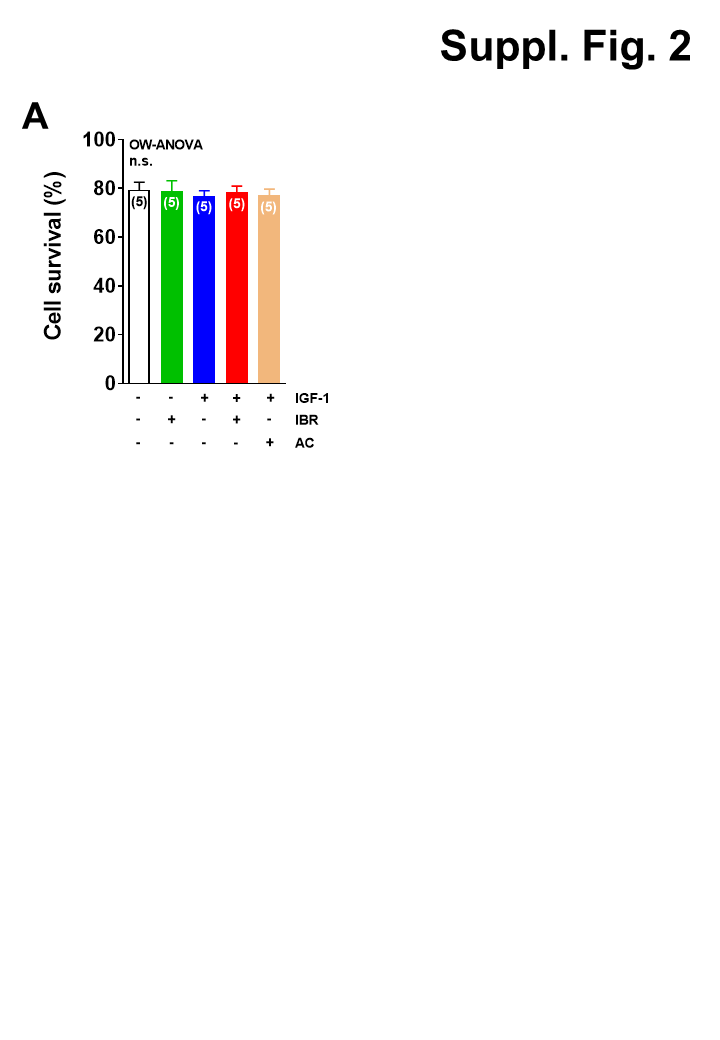

Supplement: Supplementary file 2 [file Image2.tif]
